# Exploring 6-aza-2-Thiothymine as a MALDI-MSI Matrix for Spatial Lipidomics of Formalin-Fixed Paraffin-Embedded Clinical Samples

**DOI:** 10.3390/metabo15080531

**Published:** 2025-08-05

**Authors:** Natalia Shelly Porto, Simone Serrao, Greta Bindi, Nicole Monza, Claudia Fumagalli, Vanna Denti, Isabella Piga, Andrew Smith

**Affiliations:** 1 Department of Medicine and Surgery, Proteomics and Metabolomics Unit, University of Milano-Bicocca, 20854 Vedano al Lambro, Italy; n.porto@campus.unimib.it (N.S.P.); simone.serrao@unimib.it (S.S.); g.bindi@campus.unimib.it (G.B.); n.monza@campus.unimib.it (N.M.); claudia.fumagalli@unimib.it (C.F.); isy1987@hotmail.it (I.P.); andrew.smith@unimib.it (A.S.); 2 Fondazione IRCCS San Gerardo dei Tintori, 20900 Monza, Italy

**Keywords:** 6-aza-2-thiothymine, MALDI-MSI, spatial lipidomics, FFPE

## Abstract

**Background/Objectives**: In recent years, lipids have emerged as critical regulators of different disease processes, being involved in cancer pathogenesis, progression, and outcome. Matrix-Assisted Laser Desorption/Ionization Mass Spectrometry Imaging (MALDI-MSI) has significantly expanded the technology’s reach, enabling spatially resolved profiling of lipids directly from tissue, including formalin-fixed paraffin-embedded (FFPE) specimens. In this context, MALDI matrix selection is crucial for lipid extraction and ionization, influencing key aspects such as molecular coverage and sensitivity, especially in such specimens with already depleted lipid content. Thus, in this work, we aim to explore the feasibility of mapping lipid species in FFPE clinical samples with MALDI-MSI using 6-aza-2-thiothymine (ATT) as a matrix of choice. **Methods**: To do so, ATT performances were first compared to those two other matrices commonly used for lipidomic analyses, 2′,5′-dihydroxybenzoic acid (DHB) and Norharmane (NOR), on lipid standards. **Results**: As a proof-of-concept, we then assessed ATT’s performance for the MALDI-MSI analysis of lipids in FFPE brain sections, both in positive and negative ion modes, comparing results with those obtained from other commonly used dual-polarity matrices. In this context, ATT enabled the putative annotation of 98 lipids while maintaining a well-balanced detection of glycerophospholipids (60.2%) and sphingolipids (32.7%) in positive ion mode. It outperformed both DHB and NOR in the identification of glycolipids (3%) and fatty acids (4%). Additionally, ATT exceeded DHB in terms of total lipid count (62 vs. 21) and class diversity and demonstrated performance comparable to NOR in negative ion mode. Moreover, ATT was applied to a FFPE glioblastoma tissue microarray (TMA) evaluating the ability of this matrix to reveal biologically relevant lipid features capable of distinguishing normal brain tissue from glioblastoma regions. **Conclusions**: Altogether, the results presented in this work suggest that ATT is a suitable matrix for pathology imaging applications, even at higher lateral resolutions of 20 μm, not only for proteomic but also for lipidomic analysis. This could enable the use of the same matrix type for the analysis of both lipids and peptides on the same tissue section, offering a unique strategic advantage for multi-omics studies, while also supporting acquisition in both positive and negative ionization modes.

## 1. Introduction

Among all the classes of biomolecules that have historically been the focus of studies on the mechanisms underlying human pathology, lipids have recently emerged as essential regulators of a variety of pathogenic diseases [1,2]. While these complex and heterogeneous metabolites are well-known as building blocks of membranes and energy sources, recent studies also highlighted their involvement in cancer pathogenesis, progression, and outcome [3,4,5]. Consequently, lipidomics has emerged as a new frontier research area yet to be fully explored. In this realm, mass spectrometry (MS)-based technologies represent the premier choice for the study of lipids, with their capability to comprehensively explore different sets of samples at an “omic” scale [6,7,8]. Traditional MS-based approaches have been extensively used to study biological fluids such as serum, plasma, or urine, but also tissue homogenates, in medical research [9]. However, with the advent of Matrix-Assisted Laser Desorption/Ionization Mass Spectrometry Imaging (MALDI-MSI), the technology’s reach has further expanded, making it possible to map the spatial distribution and relative abundance of analytes within tissues, including lipids [10,11]. In this context, pathology archives represent valuable biobanks that could be employed for the study of the spatial lipidome of cancer with MALDI-MSI. Formalin fixation and paraffin embedding (FFPE) is the preferred protocol for tissue preservation; however, while this method enhances histological image quality, it also depletes several lipid species during sample preparation [12,13]. As a result, the application of MALDI-MSI to FFPE samples has steadily increased in fields such as spatial proteomics and N-glycomics, but the study of lipids has mostly been hindered by this inherent loss of information. Nevertheless, recent advancements in sample preparation workflows have hinted at the possibility to still extract and map solvent-resistant lipids, principally phospholipids, which still hold biomedically relevant information [13,14]. However, MALDI matrix selection is crucial, influencing key aspects such as molecular coverage and sensitivity when seeking to maximize the information gained from this remaining lipidomic portion [15]. Common matrices include α-cyano-4-hydroxycinnamic acid (CHCA), 2′,5′-dihydroxyacetophenone (DHAP), 2′,5′-dihydroxybenzoic acid (DHB), and Norharmane (NOR) [15,16]. Several matrices proved to be efficient for spatial lipidomics on FFPE samples, but the optimal MALDI matrix for spatial lipidomics often depends on the lipid class under investigation. However, lipid analysis by MALDI-MSI is often hampered by contamination from matrix-associated clusters, which may interfere with the interpretation of mass spectra and obscure key signals from the tissue. As such, selecting a matrix that produces minimal clustering is essential. Recently, we investigated the feasibility of 6-aza-2-thiothymine (ATT) as a suitable matrix for spatial proteomics analysis [17], producing few matrix-related peaks in the low molecular weight spectral region [18]. Building on these findings, in this work, we considered the potential of ATT for lipid imaging by MALDI-MSI. As a proof-of-concept experiment, we assessed ATT’s performance for spatial lipidomics through MALDI-MSI analysis of lipids in FFPE brain sections, both in positive and negative ion modes, comparing results with those obtained from other commonly used dual-polarity matrices. DHB and NOR were chosen as comparators due to their complementary ionization properties: DHB is effective for polar lipids but can cause spectral interference, while NOR is considered a gold standard for neutral lipids with minimal signal suppression. Finally, ATT was used to perform MALDI-MSI analysis on a tissue microarray (TMA) composed of human glioblastoma cases. This final step provided an assessment of the capability of ATT to yield spatially resolved informative lipidomics data that is able to distinguish healthy and pathological conditions in clinical samples. Altogether, the results presented in this work support ATT as a possible matrix for lipidomic imaging applications, even at higher lateral resolutions (20 μm).

## 2. Materials and Methods

### 2.1. Specimen Selection

The FFPE samples utilized for this work included blocks of wild-type murine brain from male BALB/C mice sacrificed at week 16 (Ethical Approval: No. 0040933/19; Autorizzazione Ministeriale No. 169/2019-PR) and a TMA purchased from Tissuearray.com (https://www.tissuearray.com/, accessed on 30 May 2024) of human glioblastoma (GL806-L64).

### 2.2. Chemicals and Reagents

NOR was obtained from Sigma-Aldrich, Buchs, Switzerland; DHB matrix was purchased from Merck, Darmstadt, Germany; and ATT was obtained from ChemCruz, Saint Cruz, CA, USA. High-performance liquid chromatography (HPLC)-grade toluene, HPLC-grade methanol (MeOH), HPLC-grade ethanol (EtOH), and HPLC-grade water (H_2_O) were obtained from Honeywell SC, Seelze, Germany. Trifluoroacetic-acid (TFA), phosphorus red (PR), guanidinium chloride (GUA), and diammonium hydrogen citrate (DAHC) and red phosphorus were purchased from Sigma-Aldrich, Buchs, Switzerland. MMI-L Low Concentration Tuning Mix was obtained from Agilent Technologies, Santa Clara, CA, USA. EquiSPLASH™ LIPIDOMIX^®^ Quantitative Mass Spec Internal Standard was purchased from Avanti Polar Lipids (Alabaster, AL, USA).

### 2.3. Sample Preparation and Analysis

#### 2.3.1. MALDI-MS Profiling

EquiSPLASH™ LIPIDOMIX^®^ Quantitative Mass Spec Internal Standard was diluted 1:5 in MeOH and then spotted onto an MTP 384 Groundsteel target (Bruker Daltonics, Bremen, Germany) with the double-layer approach (EquiSPLASH:ATT 1:1) in triplicate (Appendix A.1).

#### 2.3.2. MALDI-MSI Analysis

To assess repeatability, three technical replicates were acquired for each matrix in both ion modes. Five micron-thick FFPE mouse brain sections were obtained and placed in triplicate on three Indium-Tin-Oxide-coated (ITO) glass slides (Bruker Daltonics, Bremen, Germany). On the day of the analysis, slides were placed in an oven at 65° for 1 h to pre-melt paraffin, and subsequently, deparaffinization was carried out with toluene washes (3 × 5 min). Then, NOR, DHB, or ATT were alternatively applied onto ITO slides with an M5 TM-Sprayer (HTX Technologies, Chapel Hill, NC, USA) according to the spraying parameters reported in Table 1 [15]. For the analysis of the glioblastoma TMA, the tissue was deparaffinized and the ATT matrix was deposited as previously described. External calibration was performed on all slides using red phosphorus.

For mouse brain imaging, a timsTOF fleX mass spectrometer was initially employed in MALDI mode, disabling trapped ion mobility spectrometry (TIMS off). TimsControl 6.0 was used to set up instrument parameters, and FlexImaging 7.5 (Bruker Daltonics, Bremen, Germany) was used to set up measurement regions for MALDI-MSI. For each matrix utilized, the left hemisphere of each brain triplicate was analyzed in reflectron positive ion mode, whereas the right hemisphere was analyzed in negative ion mode. For each analysis, a small region of the matrix was also imaged. Analyses were performed within the *m*/*z* range 400–1200. External calibration was performed using PR. A beam scan setting of 46 µm was employed in the x and y dimensions, with a raster setting of 50 µm; 400 laser shots were acquired per pixel. Moreover, to enable the tentative annotation of lipids, ion mobility separation was enabled (TIMS on) and measured in the range of 1/k0 0.70–1.80 Vs/cm^2^ to profile a representative portion of each sample for each polarity. MMI-L Low Concentration Tuning Mix was used for mobility calibration. Tune settings are reported in Appendix A.2. The number of detected peaks and the shared features across replicates are reported in Supplementary Table S3, along with the coefficient of variation (CV%) calculated for each common *m*/*z* value. Mean signal-to-noise ratios were also evaluated across replicates to support consistency in peak detection.

MALDI-MSI analysis of the glioblastoma TMA was conducted with a timsTOF fleX in TIMS off modality, utilizing reflectron positive ion mode within the *m*/*z* range 500–1100, a beam scan setting of 16 µm, and a raster setting of 20 µm. External calibration was again performed using PR. After MALDI-Imaging was performed, each tissue sample was washed (EtOH 100%, 1 × 2 min; EtOH 90%, 1 × 2 min) to remove the matrix, followed by staining with hematoxylin and eosin (H&E). The stained tissue slides were then converted to digital format using a digital scanner (NanoZoomer S60, Hamamatsu Photonics, Arese, Italy).

### 2.4. Data Analysis

#### 2.4.1. MALDI-MS Profiling

Following EquiSPLASH™ LIPIDOMIX^®^ Quantitative Mass Spec Internal Standard MALDI-MS profiling, the mean spectra of each replicate spot were visualized in Compass DataAnalysis 6.1 (Bruker Daltonics, Bremen, Germany), and peak picking was performed (SNAP algorithm, quality factor threshold: 0.9, S/N threshold: 10). Finally, peaks were matched with theoretical masses of the EquiSPLASH mixture (Table S1), considering ions [M+H]⁺, [M+Na]⁺, [M+K]⁺, and [2M+H]⁺ for positive ion mode and [M-H]^−^ for negative ion mode and adopting a mass error tolerance of 15 ppm.

#### 2.4.2. MALDI-MSI Analysis

Raw data obtained from MALDI-MSI (TIMS off) or MALDI-TIMS-MSI (TIMS on) runs of mouse brain tissue were imported into SCiLS Lab 2025b Pro (Bruker, Bremen, Germany) and normalized (Root Mean Square algorithm, RSM). For the MALDI-TIMS off datasets, peak picking was performed using the T-Rex^2^ algorithm (Weak filtering, Coverage: 50%, Rel. intensity threshold: 0.3%) and subsequently, spatial segmentation was performed (bisecting k-means algorithm; Weak denoising). Afterwards, the average spectra of the matrix and of the brain replicates were exported and loaded into mMass (v5.5.0), an open-source mass spectrometry tool, to aid spectra visualization [19]. The measurement region derived from the MALDI-MSI analysis of the glioblastoma TMA was processed correspondingly.

Afterwards, the MALDI-TIMS-MSI datasets obtained from mouse brain specimens were exported from SCiLS Lab 2025b Pro and imported into Metaboscape 2025 14.0.3 (Bruker Daltonics, Bremen, Germany) to perform tentative identification of lipids. Import parameters can be found in Appendix A.3. Tentative annotation of lipids derived from the MALDI-MSI (TIMS-off) analysis of the glioblastoma TMA was performed similarly. However, considering that ion mobility separation was not enabled, lipid annotation was solely based on *m*/*z* error and mSigma threshold. Metaboanalyst 6.0 was used to perform Student’s *t*-test (*p*-value < 0.05) and qualitative analyses (heatmap with hierarchical clustering—distance measure: Euclidean; Clustering method: Ward) [20]. LIpid Ontology (LION/web—http://www.lipidontology.com, accessed on 26 June 2025) [21] was employed to carry out lipid enrichment analysis (local statistics: 2-LOG [fold change]).

For histological mouse brain structure annotations, H&E-stained images were loaded onto DeepSlice [22] (https://www.deepslice.com.au/, accessed on 5 May 2025), a deep neural network that aligns histological sections of mouse brain to the Allen Mouse Brain Common Coordinate Framework. The output XML file was then loaded onto the QuickNII alignment tool [23] to visualize the results of the image segmentation.

## 3. Results and Discussion

### 3.1. Feasibility of ATT as a MALDI Matrix for Lipid Detection

In a first proof-of-concept MALDI-MS experiment performed on a lipid standard, ATT proved to be a feasible matrix for lipid desorption and ionization, with the most promising results obtained in positive ion mode (Table S1). In this polarity, ATT could efficiently ionize phospholipids (LPE_18.1(d7), LPC_18:1(d7), PE_15:0-18:1(d7), PG_15:0-18:1(d7), PC_15:0-18:1(d7), PS_15:0-18:1(d7), PI_15:0-18:1(d7)), sphingolipids (SM_d18:1-18:1(d9)), and ceramides (C15 ceramide-d7). Specifically, phosphocholines (PC) and sphingomyelins (SM) were the lipid classes ionized with the highest efficiency, with PC_15:0-18:1(d7), LPC_18:1(d7), and SM_d18:1-18:1(d9) reaching a respective mean relative intensity (I%) of 87.7%, 79.5%, and 68.0% amongst the three replicates, respectively, confirming the ionization tendency of these phospholipids [24]. Interestingly, no peaks could be detected for cholesterol esters (CholEster_18:1(d7)) or acylglycerols (MAG_18:1(d7), DAG_15:0_18:1(d7), TAG_15:0_18:1(d7)_15:0), although it is known that [M+Na]⁺ adducts of these species are usually detectable with other MALDI matrices, such as DHB [24,25]. This is likely due to their neutral nature and limited propensity for ionization. While this may represent a limitation in terms of broader lipidome coverage, the use of additional cationizing reagents (dopants), such as ammonium acetate, could enable the detection of TAGs, as observed with 9-Aminoacridine (9-AA) matrix [24]. Therefore, similar experiments should be conducted to enhance the detection of acylglycerols with ATT. In negative ion mode, the highest sensitivity (I%) could be achieved for phosphatidylinositols and phosphatidylserines (PI_15:0-18:1(d7): 100% and PS_15:0-18:1(d7): 19.5%), in agreement with their acidic properties. Altogether, these preliminary results confirm the capability of ATT to ionize and desorb diverse lipid classes, according to previous research conducted on oxidized phospholipids (OxPLs) [17], with satisfactory results obtained in both positive and negative polarity.

### 3.2. ATT for Lipid Detection in Mouse Brain Tissue

**Figure 1:**
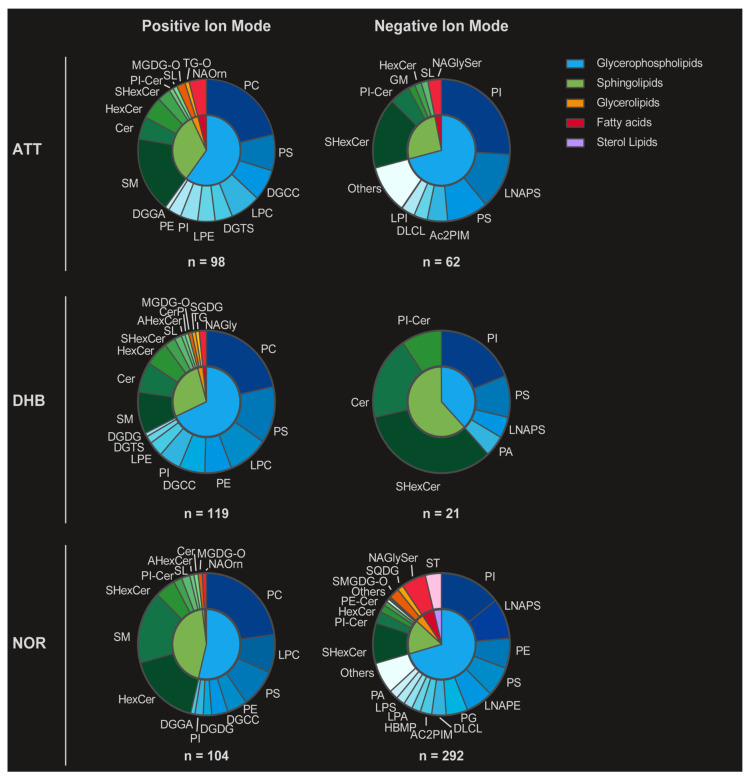
Lipid class distribution across different matrix and ionization mode conditions. Lipid abbreviations correspond to standard nomenclature (e.g., PI = phosphatidylinositol, SM = sphingomyelin, DG = diacylglycerol). Donut charts display the relative abundance of lipid classes identified with the three MALDI matrices—ATT, DHB, and NOR—analyzed in both positive and negative ion modes. Each lipid species is categorized into one of five major lipid classes: glycerophospholipids (blue), sphingolipids (green), glycerolipids (orange), fatty acids (red), and sterol lipids (purple), as indicated by the legend. Compared to DHB and NOR, ATT shows consistent performances across diverse ion modes, with a prominent detection of sphingolipids (e.g., Cer, SHexCer) in negative ion mode.

After this preliminary assessment, the applicability of ATT as a MALDI matrix on more complex biological samples was evaluated on consecutive mouse brain sections. Following MALDI-MSI analysis, tentative lipid annotation was performed, and the results were compared with those obtained with NOR and DHB in both positive and negative ion modes. Figure 1 displays pie charts summarizing the distribution of annotated lipid classes for each matrix and ionization mode, providing a visual overview of lipid class coverage. Table S2 reports the exact number and percentage of tentative annotations for each lipid class and matrix, further detailing the comparative performance in both ionization polarities. In positive mode, ATT enabled the putative annotation of 98 lipids, mostly glycerophospholipids (60.2%) and sphingolipids (32.7%), in agreement with previous results obtained on the EquiSPLASH™ standard. When comparing the results to those obtained with NOR and DHB in the same polarity, a greater number of sphingolipids could be detected with NOR (46 vs. 32) at the expense of a lower number of glycerolipids and glycerophospholipids (56 vs. 59). Conversely, DHB could enable the identification of a greater number of phospholipids (81 vs. 59). Annotation of the negative ion mode dataset led to the tentative identification of 62 lipids, of which the majority was represented by glycerophospholipids and sphingolipids (71.0% and 25.8%, respectively), similarly to the positive ion mode. In this instance, ATT could enable the detection of three times as many lipids as DHB (62 vs. 21), while NOR led to the detection of 292 lipids, the greatest number of lipids detected by a single matrix in this polarity.

The total number of unique lipid species identified under each condition is shown beneath each chart (e.g., n = 98 for ATT in positive mode). The figure illustrates distinct lipid class coverage depending on matrix and polarity, highlighting complementary detection patterns across analytical conditions. In particular, ATT allows for comprehensive lipid class representation in both ionization modes, with balanced detection of glycerophospholipids, sphingolipids, and glycolipids. Notably, ATT outperforms DHB in negative ion mode in terms of both total lipid count (n = 62 vs. n = 21) and class diversity, including key sphingolipid species such as SHexCer and PI-Cer. Although NOR yields a greater number of annotations in negative mode (n = 292), its highly complex lipid profile may introduce challenges in peak deconvolution and signal interpretation. Moreover, the repeatability of the method was confirmed by mean CV values below 30%, with 17% in positive and 27% in negative ion mode.

Interestingly, when evaluating ATT matrix for spatial proteomics [18], a low number of matrix peaks could be observed in the *m*/*z* range 700–1200, which overlaps with the typical detection range of most lipids (*m*/*z* 700–900), as shown in Figures S1 and S2. Therefore, to assess matrix peak interference in spatial lipidomics analyses, we calculated the percentage of *m*/*z* signals that could be annotated as lipids in the average spectrum for each matrix. Notably, 24.0% of the peaks in the average spectrum of mouse brain sections analyzed with ATT could be tentatively annotated, exceeding that achieved with NOR (22.9%) and DHB (13.4%). In negative ion mode, ATT enabled the annotation of 22.5% of peaks, markedly exceeding DHB (3.7%), though NOR achieved 38.8%. When looking at the S/N ratio on the replicates, ATT showed an average S/N ratio of 23 and 33 for positive and negative ion mode, respectively. These results highlight ATT as a particularly promising matrix, especially considering that NOR and DHB are already widely optimized for MALDI-MSI lipid analysis. This demonstrates ATT’s strong potential for future applications in spatial lipidomics. Importantly, ATT could represent the matrix of choice when both positive and negative ion mode analyses are desired on the same tissue section. With a single matrix application, dual-polarity measurements can be performed without the need for matrix reapplication and matrix change, simplifying the workflow. Using a single matrix for both positive and negative ion mode MALDI-MSI offers several key advantages. Firstly, it preserves valuable tissue by enabling dual-polarity analysis on the same section, which is particularly beneficial when sample availability is limited. Finally, it ensures greater spatial alignment between ion mode image acquisitions, facilitating accurate data integration and biological interpretation. This approach also reduces variability introduced by matrix-specific effects, thereby improving data consistency.

### 3.3. Feasibility of ATT Matrix for Spatial Lipidomics

Having assessed the capability of ATT to facilitate lipid ionization and desorption, the subsequent experiment was designed to evaluate its suitability for spatial lipidomics. To achieve this, consecutive mouse brain sections were analyzed in triplicate with ATT in both positive and negative ion modes, and the results were compared to those obtained with NOR and DHB. The number of features detected in the mouse brain sections varied considerably depending on the matrix utilized (Figure S1). However, the average spectra obtained with ATT exhibited fewer interfering matrix peaks in both polarities (Figures S1 and S2) in the *m*/*z* range 700–900 [26], resulting in a lower lipid signal suppression [18].

**Figure 2:**
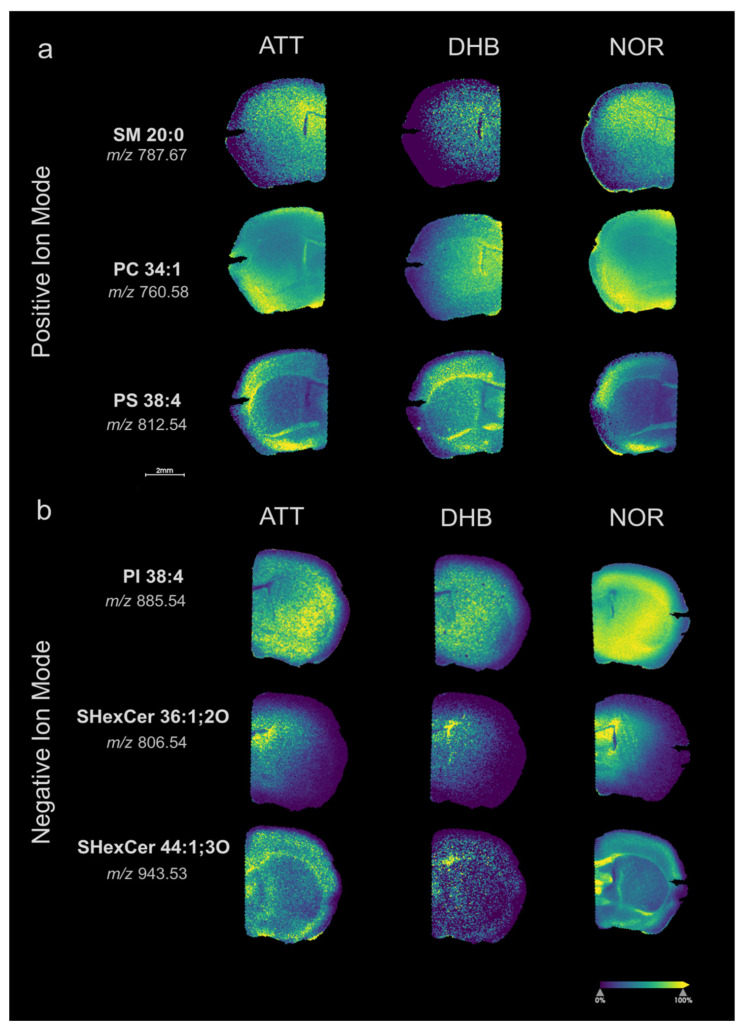
(**a**) Molecular images of three lipid species (SM 20:0; PC 34:1; PS 38:4) detected in positive ion mode with ATT, DHB, and NOR matrices; and (**b**) molecular images of three lipid species (PI 38:4; SHexCer 36:1;2O; SHexCer 44:1;3O) detected in negative ion mode with ATT, DHB, and NOR matrices. A viridis color map is reported, indicating the relative intensity of each ion from 0 (purple) to 100% (yellow).

Exemplificative molecular images, displayed in Figure 2, highlight a comparable tissue distribution of various *m*/*z* signals, irrespective of the utilized matrix.

**Figure 3:**
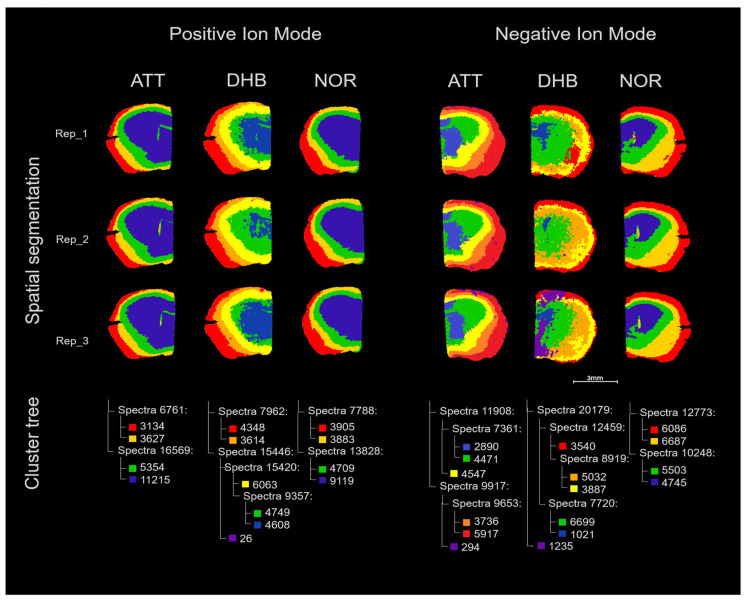
Spatial segmentation of analytical replicates of mouse brain sections analyzed with MALDI-MSI in positive and negative ion mode using ATT, DHB, and NOR matrices.

In Figure 3, the results of the spatial segmentation performed with NOR, DHB, and ATT on mouse brain sections are shown. In negative ion mode, ATT could distinguish the main histological regions of the mouse brain, including different layers of the cerebral cortex and the *caudate putamen* (Figure S3), though NOR could highlight the same regions with less branch ramifications in the cluster tree. In contrast, DHB was significantly less effective in finding clusters that corresponded to histological regions in this polarity, with only the outer layer of the cerebral cortex being clearly separated in a cluster. In positive ion mode, all three matrices were able to cluster regions corresponding to the cerebral cortex and the *caudate putamen*, with NOR and ATT being able to separate these main structures at the fourth branch ramification. Interestingly, only ATT could cluster regions corresponding to the *corpus callosum* and ventricular system at the fourth branch ramification in positive ion mode. Pearson correlation analysis of these regions revealed a high concentration of ceramides. In particular, the use of ATT markedly enhanced ceramide detection, achieving a detection rate of approximately 5%, in contrast to just 1% with NOR. While DHB showed a slightly higher detection rate (around 7%), its performance was compromised by interfering matrix-related peaks, which hindered accurate spatial segmentation. This evidence highlights ATT as particularly effective, both in terms of detection sensitivity and spatial resolution, for the investigation of ceramides. Ceramides are essential lipid molecules present in various regions of the brain, playing an important role in maintaining the integrity of the central nervous system by regulating autophagy, bioenergetics, neuronal integrity, and lipid raft signaling [27].

This dual capability of ATT, combining sensitive detection of diverse lipid species with enhanced spatial lipidomics, makes it particularly well-suited for investigating lipids such as ceramides, which exhibit complex and region-specific distributions in the brain.

### 3.4. Application of ATT to Human Glioblastoma Tissue

**Figure 4:**
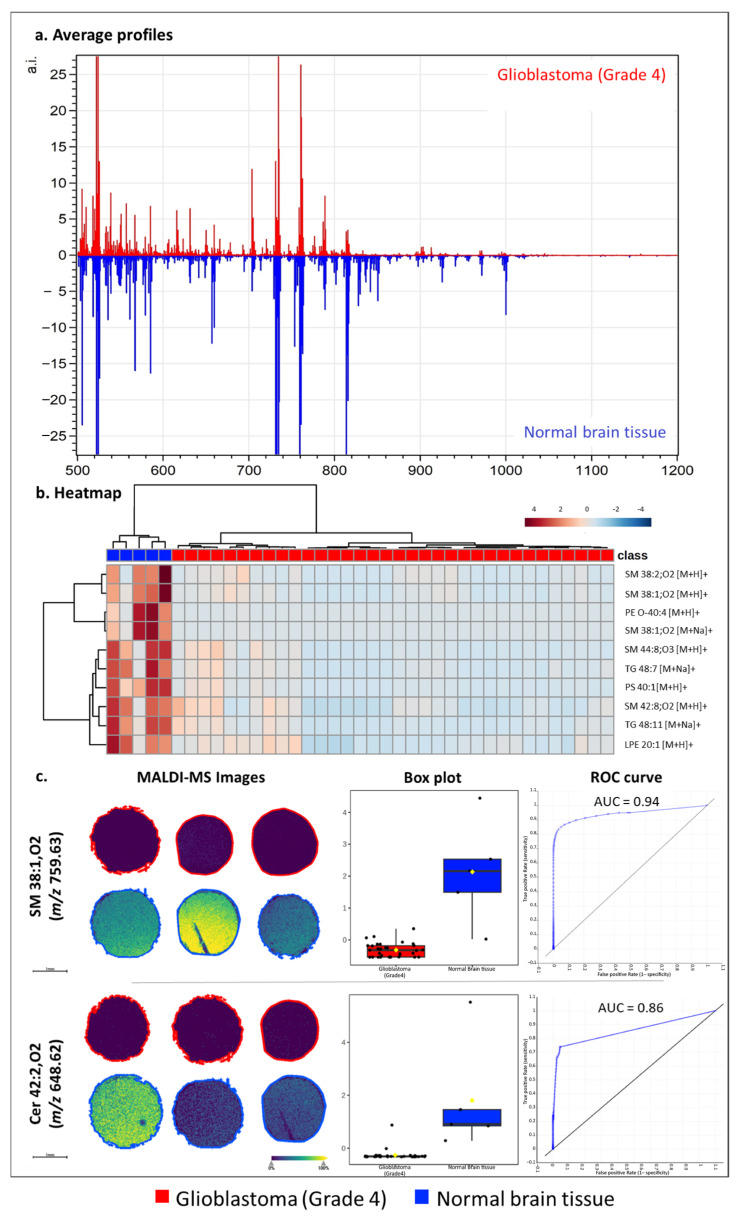
(**a**) Average lipid profiles of glioblastoma (n = 34, red) and normal brain tissue (n = 5, blue); (**b**) hierarchical clustering performed with 171 putatively annotated lipids enables the separation of glioblastoma cores (red) and normal brain tissue cores (blue). The top 10 features are shown; (**c**) Student’s *t*-test highlighted a consistent downregulation of sphingomyelin and ceramides in glioblastoma cores. Here, representative MALDI-MS images of three tumor cores and three healthy cores display the downregulation of a sphingomyelin (SM 38:1;O2) and a ceramide (Cer 42:2;O2) in tumor cores (red) compared to healthy cores (blue). An intensity box plot is also displayed, reporting mean (yellow dot) and medial value, to represent the statistically significant downregulation of these lipid species in glioblastoma, along with a receiver operating characteristic (ROC) curve demonstrating a statistically significant AUC value.

In the previous paragraphs, the suitability of ATT for spatial lipidomics in mouse brain samples was assessed, confirming its capability to ionize several classes of lipids and yielding the best results in positive ion mode. To evaluate the applicability of ATT for human pathological samples, a TMA composed of 34 glioblastoma (grade 4) cores and 5 normal brain tissue cores was analyzed under this polarity setting at a 20 μm lateral resolution. Figure 4a displays the average profiles obtained from healthy and pathological samples. The analysis led to the tentative annotation of 171 lipid features, which were utilized to perform hierarchical clustering (Figure 4b, top 10 features shown), highlighting the potential to distinguish pathological samples from healthy tissue based on the detected lipids, while also underscoring the downregulation of several lipid classes in tumor cores. Consequently, a Student’s *t*-test was performed to evaluate which lipid classes showed a statistically significant (*p*-value < 0.05) alteration amongst conditions. The analysis highlighted 60 varied putative lipids (accounting for different adducts of the same lipid). Of these, a consistent portion (17/60 = 28%) was represented by various sphingomyelins, while another important set (10/60 = 17%) was represented by ceramides and their derivatives (HexCer, PI-Cer, CerP, Table S4). To confirm these trends, an enrichment analysis was conducted, demonstrating the statistically significant downregulation of sphingomyelins and ceramides in glioblastoma samples (Table S5). Representative MALDI-MS images of a downregulated sphingomyelin (SM 38:1;O2) and ceramide (Cer 38:1;O2) are displayed in Figure 4c, along with their intensity box plots and AUC curves. Altogether, these results point to aberrant ceramide and sphingolipid metabolism, a well-known characteristic of glioblastoma. Indeed, the fine balance between sphingosine-1-phosphate (S1P) and ceramides, referred to as the “sphingolipid rheostat”, is under the lens in tumor biology due to the capacity to differentially regulate cell growth and survival [28]. This mechanism has also been linked to an altered activation of sphingomyelinases, with the enzymes involved in sphingomyelin turnover [29,30]. Specifically, in glioblastoma, a loss of growth control and a consequent gain of death resistance seem to be correlated to low levels of sphingomyelins and ceramides; this is in agreement with the results presented in this work, where the most aggressive type of glioma, glioblastoma grade 4, correlated with a downregulation of these lipid species [31]. While the thorough analysis of the sphingolipid rheostat remains outside the scope of this work, the MALDI-MSI analysis of human oncological samples with ATT could not only enable the distinction of pathological and healthy tissue but also highlight the role of a well-known lipid dysregulation mechanism in tumor growth and drug resistance.

## 4. Conclusions

In this study, we explored the feasibility of ATT as a matrix for MALDI-MSI-based spatial lipidomics on FFPE tissues. Our results demonstrate that ATT enables efficient desorption and ionization of various lipid classes, particularly phospholipids and sphingolipids, in both positive and negative ion modes. The performance of ATT was comparable to, and in some aspects, exceeded that of established matrices such as NOR and DHB, especially with respect to reducing matrix-related spectral interference and enhancing the detection of some lipid species in complex biological samples. Spatial segmentation analyses confirmed that the lipidomic information retrieved with ATT enabled the distinction of major mouse brain regions with performances comparable to NOR and improved in respect to DHB. Furthermore, the application of ATT to human glioblastoma TMA, that included both glioblastoma and healthy tissue cores, highlighted its potential to discriminate between normal and pathological tissue regions based on lipidomic profiles. Overall, the results presented in this work support the use of ATT as a viable matrix for imaging applications, not only in proteomic but now also in lipidomic analyses. In particular, ATT demonstrated good performances in the detection of polar lipids even in FFPE samples, where a substantial portion of the original lipid content is typically depleted during fixation and embedding. While, as discussed in our previous work [18], ATT’s limited solubility and vacuum stability pose some challenges, these can be mitigated through optimized protocols or the use of ambient pressure MALDI sources, supporting its future applicability in robust lipidomic and multi-omic imaging workflows.

Importantly, given its ability to support both positive and negative ion mode acquisitions, ATT could be considered a suitable matrix when dual-polarity measurements are required on the same tissue section without matrix replacement. Additionally, given the excellent suitability of ATT for spatial proteomics analysis, the use of the same matrix type for the analysis of lipids and peptides on the same tissue section may represent a strategic advantage for multi-omics investigations. Future work will focus on further optimizing the solvent composition and spray parameters to enhance the performance of ATT as a MALDI-MSI matrix for spatial lipidomic applications, with special attention paid to improving the detection of neutral lipid species. Additionally, expanding the analysis to other tissue types and pathological conditions, including different cancer models, will help evaluate the broader applicability of ATT in clinical samples.

## Figures and Tables

**Table 1:** Spraying parameters employed for each MALDI matrix.

Matrix	Conc. (mg/mL)	Solvent	Temp. (°C)	N. Passes	Flow Rate (mL/min)	Velocity (mm/min)	Pressure (psi)
ATT	10	70:30 MeOH:H_2_O 2 mM GUA 20 mM DAHC	75	4	0.12	1200	10
DHB	20	90:10 MeOH:H_2_O	60	16	0.05	1350	10
NOR	5	90:10 MeOH:H_2_O	60	16	0.05	1350	10

## Data Availability

Data will be available upon reasonable request to the corresponding author.

## Supplementary Materials

The following supporting information can be downloaded at: https://www.mdpi.com/article/10.3390/metabo15080531/s1; Table S1: (a) Lipids detected from the EquiSPLASH™ standard lipid mixture using ATT as a MALDI-MS matrix in positive ion mode; (b) lipids detected from the EquiSPLASH™ standard lipid mixture using ATT as a MALDI-MS matrix in negative ion mode; Table S2: Lipids tentatively annotated from the MALDI-TIMS-MSI analysis with ATT, DHB, and NOR in positive and negative ion mode; Table S3: Peaks detected in the brain triplicates with ATT in positive and negative ion mode. CV% and S/N ratio are reported. Table S4: Lipids species that showed a statistically significant difference (*p* < 0.05 Student’s *t*-test) between glioblastoma and normal brain; Table S5: Lion/web lipids ontology enrichment analysis. Figure S1: Average spectra obtained from the MALDI-MSI analysis of ATT, DHB, and NOR matrices confronted with the average spectra obtained from mouse brain sections analyzed with the same matrices; Figure S2: Detailed comparison of the average spectra obtained from the MALDI-MSI analysis of ATT, DHB, and NOR matrices in positive and negative ion modes; Figure S3: H&E-stained images and histological mouse brain structure segmentation and annotation obtained with DeepSlice.

## Abbreviations

The following abbreviations are used in this manuscript:

MALDI-MSI	Matrix-Assisted Laser Desorption/Ionization-MS Imaging
FFPE	Formalin fixation and paraffin embedding
DHB	2′,5′-dihydroxybenzoic acid
NOR	Norharmane
ATT	6-aza-2-thiothymine
CHCA	α-cyano-4-hydroxycinnamic acid
DHAP	2′,5′-dihydroxyacetophenone
GB	glioblastoma
TMAs	tissue microarrays
HPLC	high-performance liquid chromatography
MeOH	methanol
TFA	trifluoroacetic-acid
GUA	guanidinium chloride
DAHC	diammonium hydrogen citrate
ITO	Indium-Tin-Oxide-coated
EtOH	ethanol
MS	mass spectrometry
TIMS	trapped ion mobility spectrometry
PC	phosphocholines
SM	sphingomyelins
9-AA	9-Aminoacridine
Ac2PIM	diacylated phosphatidylinositol monomannosides
AHexCer	acylhexosylceramide
Cer	ceramides
CerP	ceramide phosphates
DGCC	diacylglyceryl-carboxyhydroxymethylcholines
DGDG	digalactosyldiacylglycerols
DGGA	diacylglyceryl glucuronides
DGTS	Diacylglyceryl trimethylhomoserines
DLCL	Dilysocardiolipins
GM	Gangliosides
HBMP	Hemibismonoacylglycerophosphates
HexCer	Hexosylceramides
LNAPE	N-acyl-lysophosphatidylethanolamines
LNAPS	N-acyl-lysophosphatidylserine
LPA	Lysophosphatidic acids
LPC	Lysophosphatidylcholines
LPE	Lysophosphatidylethanolamines
LPG	Lysophosphatidylglycerols
LPI	Lysophosphatidylinositols
LPS	Lysophosphatidylserines
MGDG-O	Ether-linked monogalactosyldiacylglycerols
MLCL	Lysocardiolipins
NAGly	N-acyl glycine
NAGlySer	N-acyl glycyl serine
NAOrn	N-acyl ornithine
PA	Phosphatidic acids
PE	Phosphoethanolamines
PE-Cer	Ceramide phosphoethanolamines
PEtOH	Phosphatidylethanol
PG	Phosphoglycerols
PI	Phosphoinositols
PI-Cer	Ceramide phosphoinositols
PMeOH	Phosphatidylmethanol
PS	Phosphoserines
SGDG	Sulfogalactosyl diacylglycerols
SHexCer	Sulfatides
SL	Sulfonolipid
SM	Sphingomielins
SMGDG-O	Semino lipid
ST	Sulfoquinovosyl diacylglycerol SQDG Cholic acids
TG	Triacylglycerols
TG-O	Ether-linked triacylglycerols

## Appendix A

### Appendix A.1

Data acquisition was carried out using a timsTOF fleX mass spectrometer (Bruker Daltonics, Bremen, Germany) and the software timsControl 6.0 (Bruker Daltonics, Bremen, Germany). For each replicate spot, 5000 spectra were acquired, respectively, in positive and negative polarity, within the *m*/*z* range 300–1500 and within 1/k0 0.70–1.80 Vs/cm^2^ ion mobility range, setting a beam scan of 100 µm and enabling random movement on the sample spot.

### Appendix A.2

Tune settings for MALDI-TIMS-MSI analysis. Parameters were set as follows for MALDI-TIMS-MSI profiling in positive ion mode: Mass range *m*/*z* 400–1200; burst of 200 laser shots; 1/k0 0.70–1.80 Vs/cm^2^; Funnel 1 RF 500.0 Vpp; Funnel 2 RF 350.0 Vpp, Multipole RF 300.0 Vpp. Parameters were set as follows for profiling in negative ion mode: Mass range *m*/*z* 400–1200; 1/k0 0.70–1.80 Vs/cm^2^; Funnel 1 RF 500.0 Vpp; Funnel 2 RF 350.0 Vpp, Multipole RF 350.0 Vpp.

### Appendix A.3

Import parameters were set as follows: Speckle size width: 3; Speckle size height: 3; Max speckles/ROI: 200; intensity threshold: 500; Abs. intensity threshold: 0.3%. Tentative lipid annotation was performed using the MS-DIAL library of authentic standards (https://systemsomicslab.github.io/compms/msdial/main.html, accessed on 10 January 2025), both in positive and negative ionization modes. Annotations were assigned, setting an *m*/*z* error threshold of 15 ppm, a CCS error threshold of 15%, and an mSigma threshold of 500.
