# Supplementary material for: Exploring 6-aza-2-Thiothymine as a MALDI-MSI Matrix for Spatial Lipidomics of Formalin-Fixed Paraffin-Embedded Clinical Samples
Source: Metabolites. 2025 Aug 5;15(8):531. doi: 10.3390/metabo15080531 (PMC12388131; doi:10.3390/metabo15080531)

Exploring 6-aza-2-thiothymine as a MALDI-MSI matrix for spatial lipidomics of formalin-fixed paraffin-embedded clinical samples

Natalia Shelly Porto [1#], Simone Serrao [1#], Greta Bindi [1], Nicole Monza [1], Claudia Fumagalli [1], Vanna Denti [1*], Isabella Piga [1,2‡] and Andrew Smith [1,3 ‡]

[1] Department of Medicine and Surgery, Proteomics and Metabolomics Unit, University of Milano-Bicocca, 20854 Vedano al Lambro, Italy; n.porto@campus.unimib.it; simone.serrao@unimib.it; g.bindi@campus.unimib.it; n.monza@camus.unimib.it; claudia.fumagalli@unimib.it; vanna.denti@unimib.it; andrew.smith@unimib.it

[2] Current address: Independent Researcher, 09121 Cagliari, Italy.; isy1987@hotmail.it

[3] Fondazione IRCCS San Gerardo dei Tintori, Monza, Italia; andrew.smith@unimib.it

[#]Equally contributing authors.

[‡]Equally co-last authors.

* Correspondence: vanna.denti@unimib.it

Figure S1: Average spectra obtained from the MALDI-MSI analysis of ATT, DHB, and NOR matrices compared with the average spectra of mouse brain sections analyzed with the same matrix. The number of peaks for each average matrix spectrum is reported. The number of lipids annotated in mouse brain with each matrix in positive and negative ion mode is reported. ATT displays fewer matrix peaks in both polarities, especially within the lipid *m/z* range (*m/z* 700-900).

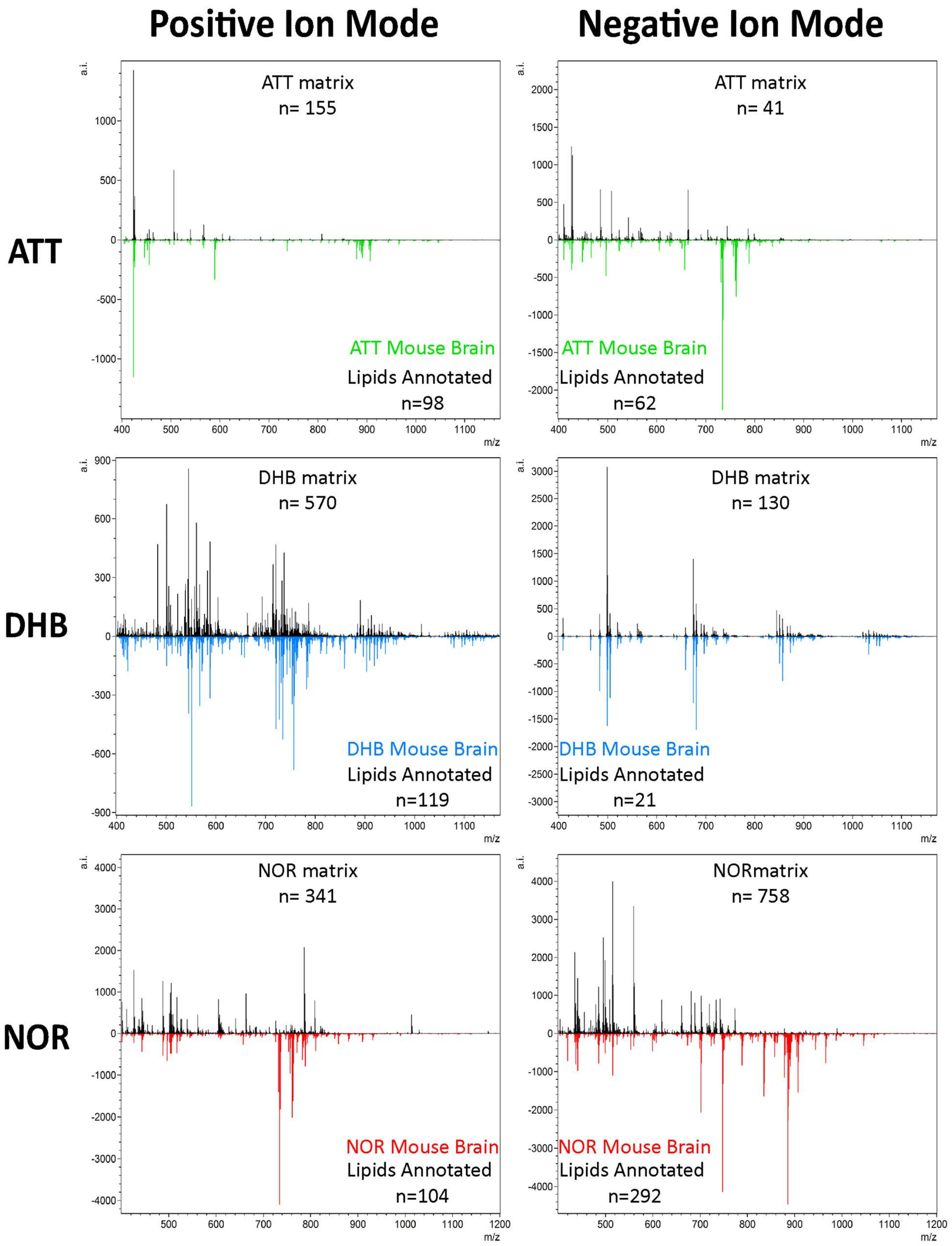

Figure S2: Detailed comparison of the average spectra obtained from the MALDI-MSI analysis of ATT, DHB, and NOR matrices, in positive and negative ion modes. The number of peaks is reported for each spectrum.

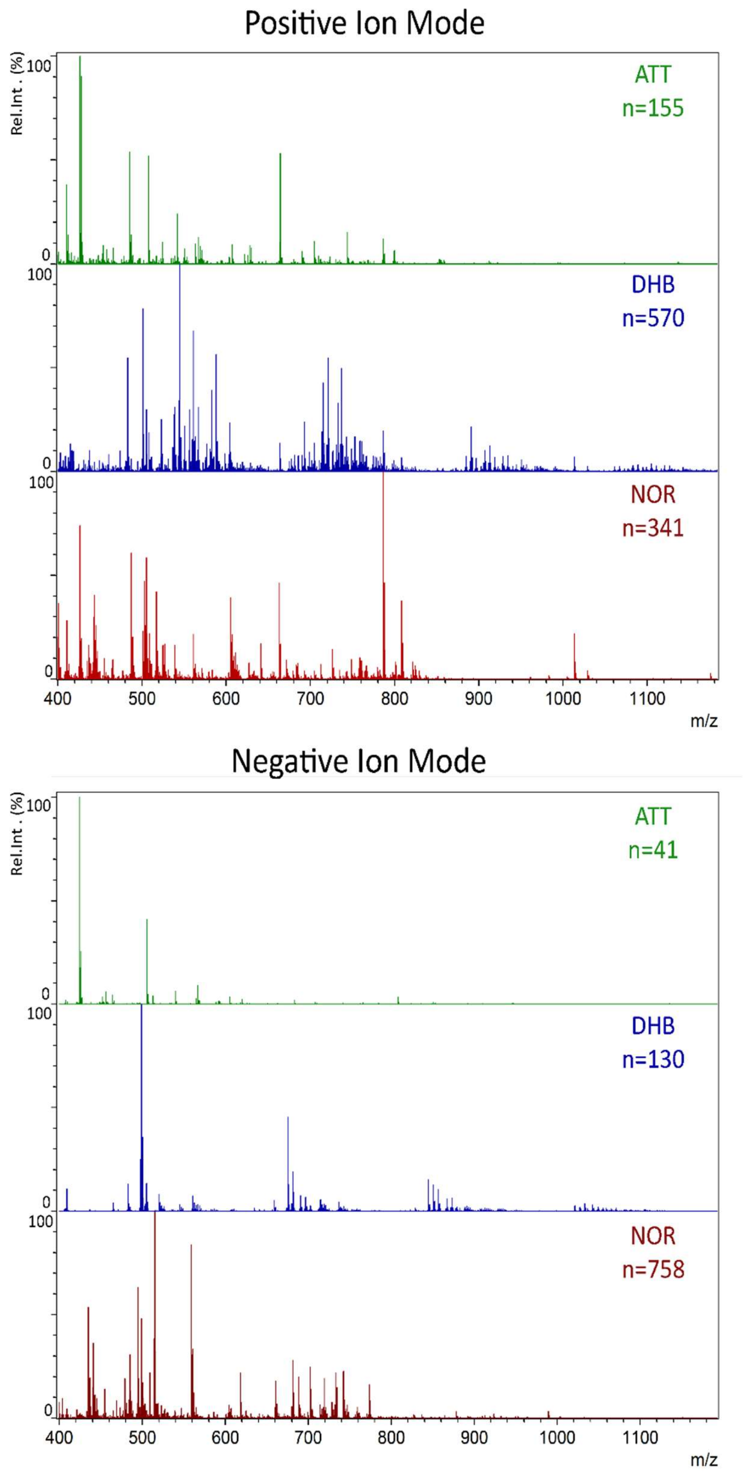

Figure S3: H&E stained images and histological mouse brain structure segmentation and annotation obtained with DeepSlice [22] (https://www.deepslice.com.au/) and visualized with QuickNII alignment tool [23]. Different colors are assigned to each histological image; those found with MALDI-MSI segmentation are also indicated with arrows and a legend

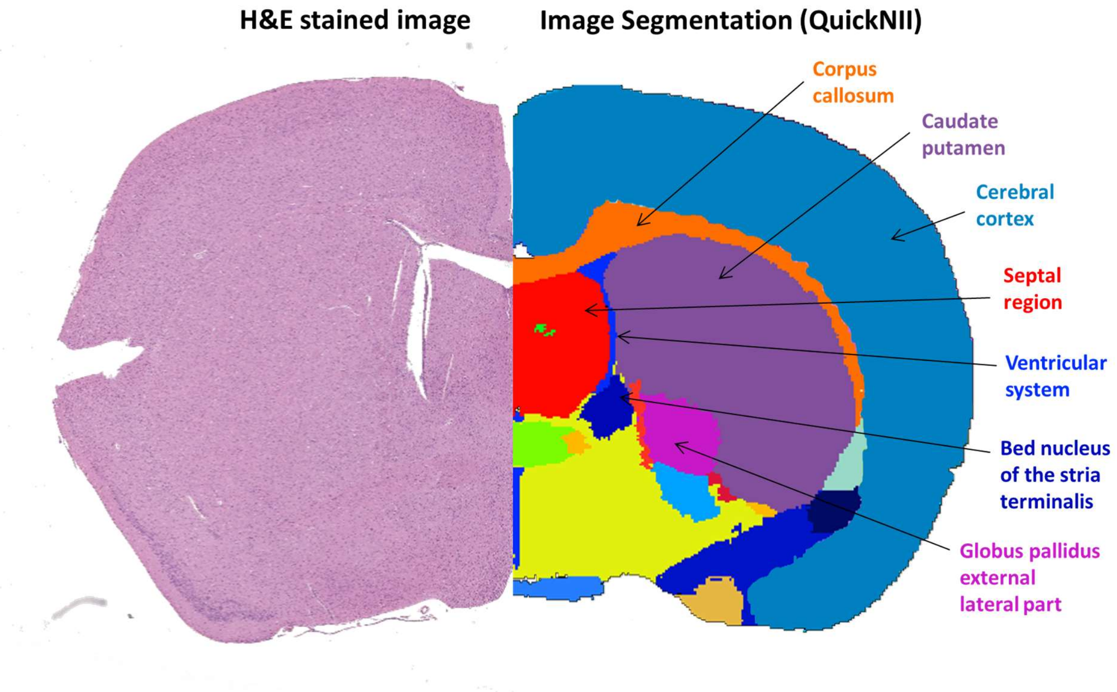

Supplement: Supplementary file 1 [file metabolites-15-00531-s001.zip › Supplementary Figures_revision.pdf]
